# Association Between Alcohol Use Disorder and Glaucoma: Evidence from the National Institutes of Health All of Us Research Program

**DOI:** 10.3390/ijerph22111738

**Published:** 2025-11-18

**Authors:** Fatima Elghazali, Alexandria N. Hughes, Gwenyth R. Wallen, Eitan Burstein, Jennifer J. Barb

**Affiliations:** 1 Translational and Biobehavioral Health Promotion Branch, National Institutes of Health, Clinical Center, Bethesda, MD 20892, USA; felghaza@uci.edu (F.E.); hughes.alexnicole@gmail.com (A.N.H.); grwallen@aol.com (G.R.W.); 2 Connecticut Eye Consultants, P.C., Danbury, CT 06810, USA

**Keywords:** glaucoma, alcohol use disorder, all of us, alcohol, alcohol abuse, alcohol misuse

## Abstract

Alcohol use disorder (AUD), defined by compulsive alcohol consumption despite harmful consequences, affected an estimated 28.1 million U.S. adults in 2023. Beyond well-known systemic effects, growing evidence suggests that alcohol may negatively influence eye health potentially contributing to the development of glaucoma, a leading cause of irreversible blindness worldwide. This retrospective cohort study evaluated the association between AUD and glaucoma in a large, diverse population using data from the National Institutes of Health (NIH) All of Us Research Program. Multivariable logistic regression, adjusting for sociodemographic confounders, was applied to assess glaucoma diagnoses among participants with and without AUD. Adults (*n* = 122,706) with a mean age of 56.7 years (SD = 16.8) and 66% female were assessed. Individuals with AUD had significantly higher odds of a glaucoma diagnosis compared with those without AUD (odds ratio: 1.45; 95% confidence interval: 1.35–1.57; *p* < 0.001). These findings suggest that AUD may be an underrecognized risk factor for glaucoma and that preventative care for eye health may be warranted in this population. Additional screening in higher-risk individuals may improve long-term quality of life and reduce the broader public health burden of glaucoma.

## 1. Introduction

Alcohol use disorder (AUD) is a condition characterized by uncontrollable consumption of alcohol despite negative social, occupational, or health consequences [1]. According to the National Survey on Drug Use and Health, 28.1 million adults ages 18 and older had AUD in 2023 [2]. The Diagnostic and Statistical Manual of Mental Disorders 5th Edition (DSM-5), which was updated in 2013, defines AUD as a disorder that encompasses alcohol abuse and alcohol dependence [3]. Prior to this release, several different terms were used to describe a high level of alcohol consumption, including alcohol abuse, alcohol dependence, and others. Importantly, no amount of alcohol consumption is considered risk-free, and alcohol contributes to over 3 million deaths annually worldwide, as reported by the World Health Organization (WHO) [4]. AUD is linked to an increased risk of developing noncommunicable diseases, including liver and heart disease, various cancers, and mental and behavioral conditions [4]. However, chronic alcohol consumption affects most major organ systems, including the eye and ocular physiology [5,6,7]. Yet, the relationship between AUD and the development or co-occurrence of glaucoma, a significant eye disease and cause of visual disability, remains unclear.

Glaucoma is a progressive eye disease causing vision impairment and eventual blindness. With no known cure, glaucoma is the leading cause of irreversible blindness worldwide, affecting approximately 4.2 million Americans and 80 million people globally as of 2020 [8]. Although it can affect individuals of all ages, older adults and postmenopausal women appear to be at higher risk [8,9,10]. Chronic alcohol use or AUD is often associated with elevated intraocular pressure (IOP), a risk factor for glaucoma development and progression, although glaucoma can also occur with normal IOP [11,12,13]. Acute alcohol consumption levels have been found to transiently lower IOP due to isosmotic properties, although chronic alcohol use may raise IOP, suggesting a dose-dependent adverse association between alcohol and IOP [13,14] [Song, J.E., et al.], Effects of Consumption 2020 [9]. Alcohol use is also known to be a risk factor for systemic hypertension, which is a risk factor for increased IOP [Mahmoudinezhad 2023; Grant A 2023] [12,13]. Additionally, glaucoma is a type of optic neuropathy characterized by optic nerve degeneration [Bussel, II and A.A. Aref, Dietary factors 2014] [15]. In the short-term, alcohol use alters the net amount of water in the aqueous humor as well as the production, thus affecting the flow of the aqueous humor and IOP regulation [11]. Alcohol use is also known to be a risk factor for systemic hypertension, which is a risk factor for increased IOP [14,15]. Additionally, glaucoma is a type of optic neuropathy characterized by optic nerve degradation [16], and chronic heavy alcohol use is a known cause of neuropathy through oxidative stress and optic nerve damage, which in turn may worsen glaucomatous damage [17,18]. Cannabis has been studied as a potential treatment for glaucoma since the 1970’s [19]. Cannabinoids exert a short-term lowering of IOP by reducing aqueous humor production and promoting outflow [19]. Taken together, these mechanisms suggest that AUD or chronic alcohol abuse may set the stage for glaucoma development, with a potential mitigating effect of cannabis.

Current epidemiological evidence on the association between alcohol consumption and glaucoma presents mixed findings. Some studies have reported no association, while others have indicated alcohol may act as either a protective factor or risk factor for glaucoma [17,20,21]. A cross-sectional observational study examining the association between alcohol consumption with glaucoma and related traits revealed that alcohol intake was consistently adversely associated with glaucoma and related risk factors at levels below current national drinking guidelines [13]. Another study, a retrospective cohort study conducted in Japan from a large-scale administrative claims database reported that consuming ≥180 mL (2.5 units) of alcohol per day was associated with an increased risk of glaucoma compared to lower consumption whereas moderate daily alcohol intake was linked to a decreased risk [17]. Similarly, a protective association was found between alcohol use and the development of primary open-angle glaucoma (POAG) in a small hospital-based Chinese cohort [20]. By contrast, a large prospective cohort study examined alcohol consumption and the incidence of exfoliation glaucoma/glaucoma suspect (XFG/XFGS) status and found that greater alcohol consumption was associated with a significantly higher XFG/XFGS status risk [22]. Although several studies have investigated the association between alcohol use and glaucoma, the conclusions remain inconsistent. Some studies present a positive association, others suggest a protective effect, while some find no association at all. The varied findings from these studies may be due in part to differences in study design, limitations including the varied cohort sizes and settings, focus on differing glaucoma subtypes, how alcohol exposure is measured, and adjustment for different covariates in multivariable models. Furthermore, these studies did not specifically assess the risk among individuals with clinically diagnosed AUD. To address these gaps, the aim of this current study is to determine primary glaucoma prevalence in individuals with AUD using data from a large, observational cohort study. We selected the National Institutes of Health (NIH) All of Us (AoU) Research Program to test the hypothesis that AUD would be associated with glaucoma development because NIH AoU aims to represent the diversity of the United States and contains electronic health records (EHR) and surveys that collect clinical diagnoses from participants. This study aims to clarify the direction and strength of the association between clinically diagnosed AUD and glaucoma in a large national database.

## 2. Materials and Methods

### 2.1. Study Population Characteristics

This study includes data from the NIH AoU Research Program to test the hypothesis that AUD or AUD related diagnoses are a risk factor for glaucoma diagnosis. We used electronic health records (EHR) and patient-reported health histories to establish the presence of AUD diagnoses and glaucoma diagnoses and drew upon AoU surveys to determine demographics and lifestyle habits. The AoU Research Program is a nationwide initiative focused on increased sample diversity with a goal of enrolling over one million participants. At the time of the data access (October 2024), there were 413,457 participants enrolled in the AoU Controlled Tier v7 release data. The v7 release contains data that spans until 1 July 2022 and extends as far back as is available via Health Care Provider Organization-sourced EHR, which may vary by organization. A complete characterization of the data contained is available at: https://support.researchallofus.org/hc/en-us/articles/14558858196628-2022Q4R9-v7-Data-Characterization-Report (accessed on 25 October 2025). All participants gave written informed consent at the time of enrollment, and the study was approved by the NIH AoU Institutional Review Board. The AoU Researcher Workbench employs a data passport model, through which authorized users do not need IRB review for each research project. Therefore, this study will not be conducting human subjects research with All of Us data as the research does not directly involve participants, only their data and the data available in the workbench has been thoroughly checked and edited to remove identifying information while assuring its use for scientific research. The program gathers extensive data from participants, including physical measurements, EHR, survey responses, wearable device data, and biospecimens. The cohort was limited to participants that had volunteered to share their EHR data with AoU. Shared EHR is not guaranteed to be a complete lifetime record and may not include EHR from Health Care Provider Organizations that do not share data with AoU.

**Figure 1:**
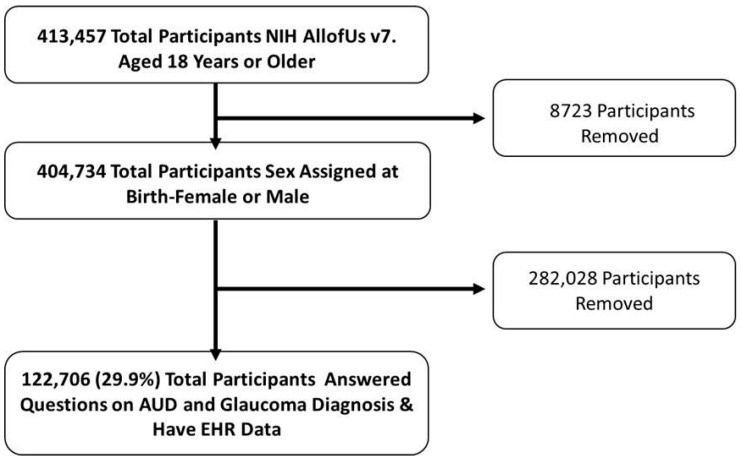
Schematic of Participant Inclusion using NIH AoU Research Database. Legend: Inclusion and exclusion criteria flow chart for the current study cohort. Individuals 18 years or older and sex assigned at birth (male or female) were included. Total participants included in the cohort based on AUD and glaucoma diagnoses determined by EHR data and questionnaires.

Participants who were at least 18 years old, had a sex assigned at birth of male or female, had opted in to contributing EHR data, and who answered questions covering glaucoma and AUD diagnoses on the Personal & Family Health History Survey were included. Overall, in the v7 database, there were 413,457 participants aged 18 years or older, of which 404,734 total participants were assigned male or female sex at birth, of which 122,706 (29.9%) were identified to have information on glaucoma and AUD diagnoses (Figure 1).

### 2.2. Identifying Glaucoma and Alcohol Variables 

The outcome of interest for this analysis was diagnosis with a primary glaucoma condition in the imported EHR or identified in the Personal & Family Health History survey; participants with secondary glaucoma and congenital types of glaucoma were excluded. The exposure variable was an alcohol abuse related condition which included self-reported AUD, and a myriad of physiological conditions resulting from chronic alcohol misuse. From here and throughout, these will be described as AUD diagnoses. The EHR-included conditions related to AUD, indicating alcohol abuse or dependence, and all conditions included are shown in Supplementary Table S1. To determine which participants had alcohol abuse-related conditions, as a proxy for AUD, we filtered all EHR conditions including the term “alcohol” that implied alcohol dependence and utilized the Personal & Family Health History survey to further gather participants who reported previously being diagnosed with AUD but may not have had the condition included in the EHR. Complete lists of conditions extracted from the EHR data for both the outcome and exposure variables are shown in Supplementary Tables S1 and S2. Additionally, 2631 and 2398 participants, respectively, disclosed AUD and glaucoma diagnoses in the Personal & Family Health History Survey, however these diagnoses were not included in imported EHR, so precise diagnoses are not tabulated. 

### 2.3. Covariates

Covariates were selected a priori based on review of the literature and included age, sex assigned at birth, body mass index (BMI), smoking status, and cannabis use. Age was computed as the difference between participant date of birth and the AoU v7 release date of 1 July 2022. Sex assigned at birth was provided in the prepackaged *Demographics* file and the calculated BMI was available in the *Physical Measurements* data. Smoking status was defined as current smoker, ex-smoker, or never smoker using survey questions with concept ids #1333011 (“In the past month: Did you smoke tobacco/nicotine (including cigarettes, cigar, cigarillos, pipes, hookah) every day, some days, or not at all?”), #1585857 (“Have you smoked at least 100 cigarettes in your entire life? (There are 20 cigarettes in a pack.)?”), and #1585860 (“Do you now smoke cigarettes every day, some days, or not at all?”). Participants were defined as cannabis users or non-users using survey questions with concept ids #1585636 (“In your LIFETIME, which of the following substances have you ever used?”—Marijuana use) and #1333017 (“In the past month, have you used any of the following drugs? Select all that apply.”—Cannabis). Multiple questions were sourced to ensure coverage of all participants in the cohort. 

### 2.4. Description of AoU Data Access

AoU Controlled Tier v7 release data in the Researcher Workbench were accessed in accordance with the NIH Clinical Center’s Data Use Agreement. The analytic dataset specifications were created using the Cohort Builder and Dataset Builder, and the resulting query was run in R version 4.4.0 in RStudio in the Researcher Workbench with the package bigrquery to create dataframes containing selected data for our defined cohort (AoU Workbench function). This research does not require ethical approval, although our research adheres to the ethical principles upheld by the AoU research program. 

### 2.5. Statistical Analysis

To determine if any sex differences were present between covariates assessed in our cohort, statistical testing was performed using *t*-tests for continuous variables and chi-square tests for categorical variables. Statistical testing of sex differences between covariates was carried out via the ‘Tableone’ package in R (CRAN project). For the main model, multivariable logistic regression was conducted in R using the ‘glm’ function with the family set to “binomial” to fit a generalized linear model. The outcome variable was glaucoma diagnosis, and the exposure was AUD diagnoses, adjusted for covariates age, sex assigned at birth, body mass index (BMI), smoking status, and cannabis use. Missing data were minimal across all variables (4.2%) and a complete case analysis was performed on n = 117,578 participants with complete data for the outcome, exposure, and all covariates. For model diagnostics, deviance residuals were examined, and standardized generalized variance inflation factors (GVIF) were examined to check for multicollinearity that could violate assumptions underlying hypothesis testing. All terms had standardized GVIF values between 1.01–1.07, indicating a near absence of multicollinearity. For classification performance area under the receiver operating characteristic curve was computed using the ‘pROC’ package in R. Coefficient estimates and 95% profile confidence intervals were exponentiated to report results on the odds scale.

## 3. Results

### 3.1. Participant Characteristics

**Figure 2:**
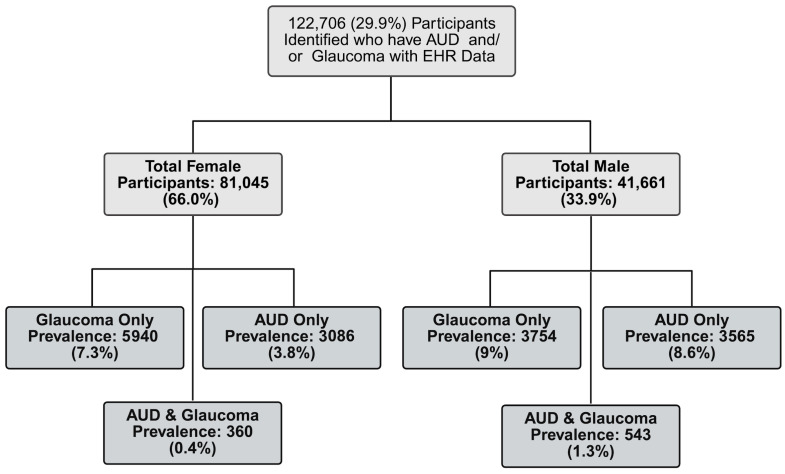
Prevalence of Glaucoma and AUD for Females and Males in Cohort. Legend: Prevalence rates between male and female participants across the entire cohort, stratified by AUD and glaucoma diagnoses.

Of the 122,706 participants included in this cohort, 81,045 (66.1%) were female and 41,661 (33.9%) were male (Table 1). The mean age of participants was 56.7 years (SD = 16.8), and the mean BMI was 29.6 (SD = 7.4). A total of 10,597 participants (8.6%) had a glaucoma diagnosis, while 7554 (6.2%) had an AUD-related diagnosis. All demographic characteristics showed significant differences by sex (Table 2). Compared to women, men were older, had a slightly lower average BMI, and exhibited a higher prevalence of both glaucoma and AUD. Men were also more likely to be current smokers and to report prior cannabis use. In our cohort, men had a higher prevalence of both glaucoma and AUD, while females had a slightly lower prevalence rate (Figure 2). 

### 3.2. Prevalence of Glaucoma Diagnoses and AUD-Related Diagnoses Across Study Cohort 

A combined total of 7554 individuals had AUD, of which 4923 (65.2%) had an AUD related condition in imported EHR, and 2631 (34.8%) self-reported receiving a previous AUD diagnosis that was not contained in the EHR made available to AoU. EHR conditions that qualified as AUD diagnoses are tabulated in Table 3. The most frequent conditions were alcohol abuse, alcohol dependence, chronic alcoholism in remission, alcohol withdrawal syndrome, and alcoholic cirrhosis (Supplementary Table S1). A combined total of 10,597 individuals had a glaucoma diagnosis, of which 8199 (77.4%) had a glaucoma condition in imported EHR, and 2398 (23.4%) reported a previous glaucoma diagnosis that was not available within AoU. The most prevalent conditions were borderline glaucoma, glaucoma, and different types of open-angle glaucoma (Supplementary Table S2). Of the 7554 participants with AUD, 6651 (5.4%) had only AUD, while 9694 (7.9%) had glaucoma only. A total of 903 (0.73%) participants had both AUD and glaucoma. 

### 3.3. Assessing the Association of Glaucoma Diagnosis with AUD-Related Diagnoses

First, we assessed univariable relationships between glaucoma diagnosis and AUD diagnosis and each of the participant covariates selected a priori, which included participant age, sex assigned at birth, BMI, smoking status, and history of cannabis use. In univariable logistic regression models of glaucoma diagnosis, AUD diagnosis as well as all covariates except BMI were significantly associated with glaucoma diagnosis (Table 3). AUD diagnosis, age, male sex, and a history of smoking were each associated with an increase in the odds of glaucoma diagnosis, whereas previous cannabis use was associated with decreased odds of glaucoma diagnosis.

**Figure 3:**
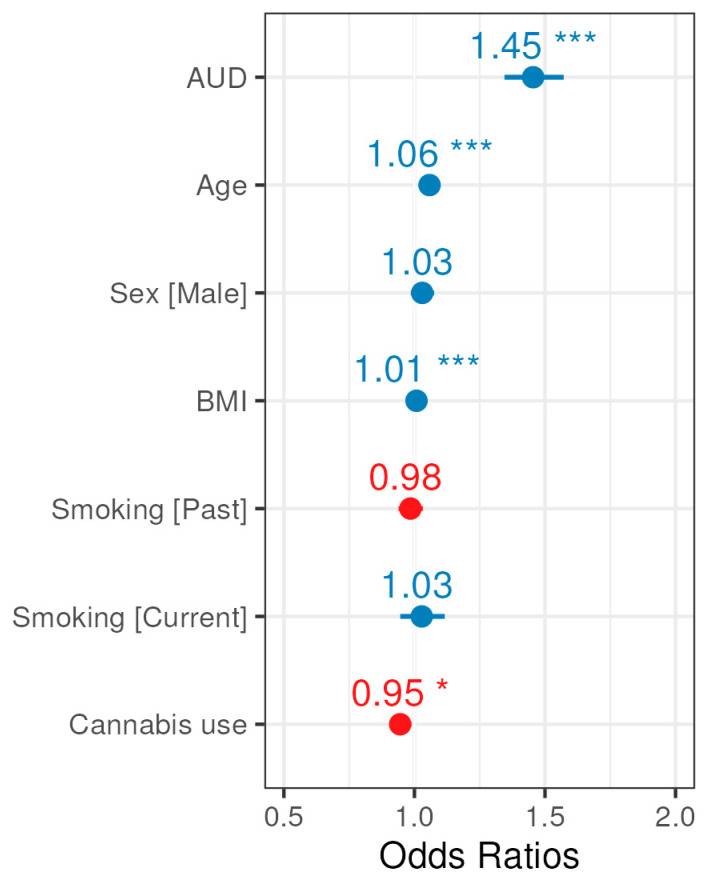
Forest plot for odds ratio estimates from the glaucoma multivariable logistic regression model. Blue indicates a positive association with glaucoma, whereas red indicates a negative association, and asterisks (*) denote significance (*p*-values provided in Table 4).

Next, multivariable logistic regression was used to model glaucoma diagnosis with AUD diagnosis as the exposure, adjusted for participant covariates. Adjusting for age, sex assigned at birth, BMI, smoking status, and cannabis use, individuals with any AUD diagnoses had a 45% increase in the odds of glaucoma diagnosis relative to individuals without AUD (OR 1.45, 95% CI: 1.35–1.57, *p* < 0.001), (Table 4 and Figure 3). Furthermore, smaller significant effects were observed for Age, BMI, and history with cannabis use. 

**Figure 4:**
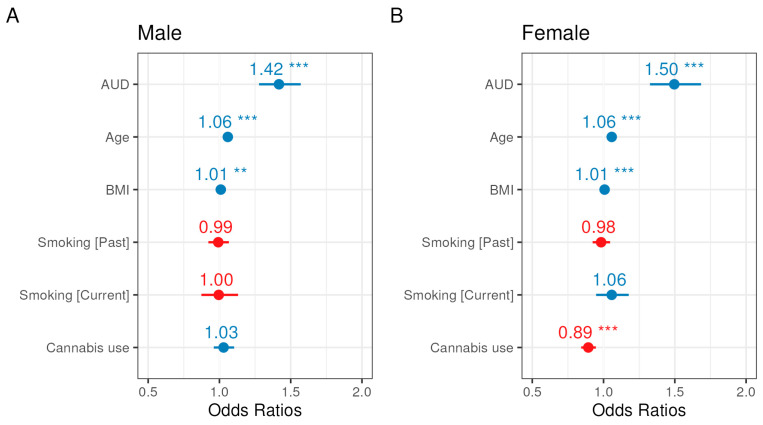
Forest plots for odds ratio estimates from the sex-stratified glaucoma multivariable logistic regression models for men (A) and women (B). Blue indicates a positive association with glaucoma, whereas red indicates a negative association, and asterisks (*) denote significance (*p*-values provided in Supplementary Tables S4A and S4B).

Because all covariates were significantly different between the sexes (Table 2), we additionally performed statistical modeling with (1) an AUD and sex interaction term to evaluate possible effect modification by sex, and (2) stratified models by sex. The relationship between AUD and glaucoma was not modified by sex, evidenced by a non-significant interaction term (Supplementary Table S3), and sex-stratified models yielded similar odds ratios for covariates and the exposure (Figure 4). However, the reduced odds of glaucoma diagnosis among cannabis users held in the female-stratified model, but the odds were not reduced among cannabis users in the male-stratified model (Figure 4, Supplementary Tables S4A and S4B).

We undertook an additional sensitivity analysis to determine whether the relationship between glaucoma and AUD diagnoses depended on the source information for AUD diagnosis, because our main analysis pooled participants with EHR-derived and self-reported AUD diagnoses. Specifically, we fit multivariable logistic regression models for glaucoma diagnosis similar to the main model shown in Table 4, but rather than pooling EHR-derived and self-reported AUD diagnoses, we separately evaluated the relationship that each independent source had with glaucoma diagnoses. Both EHR-derived and self-reported AUD diagnoses were significantly associated with glaucoma diagnosis, with a stronger relationship evident between EHR diagnoses (OR: 1.70, 95% CI: 1.55–1.85, *p* < 0.001) than self-reported diagnoses (OR: 1.22, 95% CI: 1.10–1.36, *p* < 0.001) (Supplementary Tables S5A and S5B). Model classification performance as determined by area under the receiver operating characteristic curve (AUC-ROC) was similar between the main model, the model in which only self-reported AUD diagnoses were used, and the model in which only EHR-derived AUD diagnoses were used (all ~0.72) (Supplementary Table S6).

## 4. Discussion

In this study, we used data from the version 7 release of the NIH All of Us (AoU) Research Program to examine the association between AUD and glaucoma diagnoses among 122,706 participants from a large, real-world population. This release reached 40% of the recruitment target for the AoU program [23]. A central goal of the AoU Research Program is to provide a repository of data that is reflective of the rich diversity of the United States population and our findings benefit from the deliberate efforts by the program to make participation widespread and inclusive [24]. After adjusting for demographic and lifestyle covariates, we found that individuals with AUD or AUD related diagnoses had 45% higher odds of having a glaucoma diagnosis (OR = 1.45; 95% CI: 1.35–1.57) after adjusting for covariates compared to those without an AUD diagnosis indicating a significant association between the two diagnoses (Table 4, Figure 3). This large increase in the odds of glaucoma among individuals diagnosed with AUD did not depend on sex (Figure 4, Supplementary Tables S4A and S4B) and remained evident when EHR-derived and self-reported AUD diagnoses were separately evaluated, indicating that this relationship is robust to different reporting measures (Supplementary Tables S5A, S5B and S6). However, we caution that as a cross-sectional analysis, the strong relationship we detected between AUD and glaucoma is an association that will require further longitudinal study to determine causality.

The prevalence of AUD in our cohort was consistent with national estimates. According to the United States 2023 National Survey on Drug Use and Health, AUD prevalence is higher among males (12.1%; 16.8 million) than females (8.3%; 12 million) [2]. Our cohort demonstrated a similar pattern, with a higher prevalence of AUD among males (9.9%; 4108) compared to females (4.3%; 3446) (Figure 2). Glaucoma, which affects an estimated 4.22 million U.S. adults, also shows a slightly higher prevalence in men (1.65%) than women (1.59%) [9]; our cohort similarly had a higher prevalence among men than women, but a higher overall prevalence in both sexes than the national estimates (Figure 2). Due to sex-based differences in prevalence and key risk factors for glaucoma, we investigated whether the relationship between AUD and glaucoma would differ between men and women. While we did not identify a significant effect modification by sex, we did find that the main result for slightly lower odds of glaucoma diagnosis among cannabis users was present in women but not men (Figure 4). We adjusted for cannabis use in our models because previous work has indicated potential effects on IOP [19]. However, this result for cannabis is based upon the limited, self-reported history of prior use information available in the AOU lifestyle survey and warrants further investigation in a setting that can account for more details of use.

Our finding that AUD is associated with glaucoma diagnosis is consistent with estimates from studies performed in other cohorts that have examined excessive alcohol intake. A large prospective cohort study found that participants consuming ≥15 g/day of alcohol had a 55% higher multivariable rate ratio (MVRR, 1.55; 95% CI, 1.17–2.07) for exfoliation glaucoma (XFG/XFGS) compared with nondrinkers (*p* = 0.02 for trend) [22]. Notably, this association was also observed for glaucoma in a cross-sectional analysis of UK Biobank data at alcohol intake levels below the current U.S. guidelines, defined as less than 98 g per week for women and less than 196 g per week for men, in which former drinkers were found to have a higher prevalence of glaucoma (OR 1.53; *p* = 0.002) [13]. Other cohort studies have found smaller increases in risk, including one involving 3.1 million participants from Japan that found that consuming ≥2.5 units of alcohol per day was associated with a 5–6% increased risk of glaucoma compared with consuming <2.5 units/day (HRs ranging from 1.05–1.06; 95% CIs: 1.01–1.12) [17]. Importantly, reducing alcohol consumption may prevent glaucoma progression. A retrospective analysis of Korean National Health Insurance data found that abstaining from alcohol after glaucoma diagnosis was associated with a 37% reduction in risk of visual impairment (AHR = 0.63) compared to newly diagnosed individuals who continued to consume alcohol, who had higher risk of progressing to vision loss (AHR = 1.78–2.56) [25]. Consistent with the possibility that continued consumption worsens disease, a retrospective cohort study of adults with suspected glaucoma identified alcohol intake as a risk factor for subsequent glaucoma diagnosis [14]. Although clinical recommendations have not yet been established, one analysis suggested that limiting alcohol intake to fewer than 2.5 units per day may reduce glaucoma risk [17].

While some studies have shown a strong association with alcohol use and increased risk of glaucoma, other studies have shown inconsistent associations. In a study investigating the risk factors of primary-open angle glaucoma, using multivariate logistic regression, investigators concluded alcohol consumption may have a protective effect against primary open-angle glaucoma, evidenced by a strong negative association and low odds ratio [20], while others found no significant association [15,26,27]. These discrepancies may stem from differences in cohort characteristics, methods of quantifying alcohol intake (e.g., grams per day vs. categorical drinker status), and the covariates included in statistical models. Overall, our results support the role of chronic alcohol misuse as a risk factor for glaucoma, but as a cross-sectional study, we also highlight the need for more longitudinal research in this area. 

Mechanistic studies have investigated the effect of alcohol on key glaucoma-related markers, and risk factors such as high IOP and retinal structures. For example, IOP has been found to be elevated in daily drinkers, defined as drinking six or more times per week, compared to non-drinkers [15], and excessive alcohol intake has been associated with thinner retinal nerve fiber layer (RNFL) and ganglion cell–inner plexiform layer (GCIPL) thicknesses [28,29]. Oxidative stress, an established consequence of extended alcohol exposure, may damage retinal ganglion cells and trabecular meshwork activity, both of which play a part in the pathogenesis of glaucoma [30]. Glaucoma is characterized by the loss of retinal ganglion cells, and the RNFL and GCIPL are used to measure and detect glaucoma and glaucomatous damage [31]. Furthermore, prior studies have examined dietary intake and the relationship between vitamins and glaucoma, identifying beneficial associations between higher vitamin A and vitamin C intake and reduced risk of open-angle glaucoma [26]. In contrast, alcoholism or AUD often results in to nutritional deficiencies, stemming from impaired gastrointestinal absorption and reduced nutrient intake, which can increase the risk ofdeveloping nutritional optic neuropathy [32].

This study leveraged data from the All of Us (AoU) Research Program, which provided a large and demographically diverse cohort of adults, with broad representation across age, sex, and race/ethnicity. The inclusion of key lifestyle covariates, such as smoking status and cannabis use, was strengthened by a high survey response rate, with 95.8% of participants completing the relevant surveys. Additionally, the integration of EHR data with participant-reported information enhanced the depth of the dataset and enabled us to estimate the prevalence of glaucoma among participants with AUD. 

### Limitations of the Study

Despite these strengths, several limitations warrant discussion. Although we sought to examine additional glaucoma-related risk factors, such as IOP, vitamin lab values (e.g., deficiencies or excesses), and ophthalmologic imaging and exam data (e.g., optical coherence tomography, pachymetry, and visual field testing), these data were largely unavailable or incomplete. While EHR entries indicated that such assessments were performed, actual measurement values or sufficient detail for meaningful analysis were often missing. For example, fewer than 0.01% of participants had available data on IOP or relevant vitamin levels, precluding our ability to evaluate their roles in the observed association between AUD and glaucoma. Additionally, the study’s reliance on voluntary participation and observational data introduces the possibility of misclassification and underreporting, particularly for self-reported variables such as alcohol and cannabis use. Information on cannabis use was limited to survey questions inquiring about any recent use, without capturing motivation for use, frequency or quantity. Finally, the cross-sectional nature of the analysis restricted causal inference, and we are only able to conclude that there is an association between AUD and glaucoma. Future research with prospective designs and more complete clinical data may help clarify the biological mechanisms linking AUD to glaucoma risk. 

Globally, an estimated 400 million individuals, or 7% of the earth’s population ages 15 years or older have alcohol-related disorders, and an estimated 209 million, 3.7% of the adult global population have alcohol dependence according to the WHO’s global status report in 2024 [33]. Alcohol is a known modifiable risk factor for glaucoma, making it essential for individuals to make informed decisions regarding alcohol consumption and to discuss intake with their healthcare providers [13]. This study adds to existing public health knowledge, aiming to improve health outcomes and interventions for at-risk populations, such as those with AUD. The findings may serve as a resource for advocacy and the development of integrative whole person preventative care strategies in this high-risk population.

## 5. Conclusions

This large cohort study found that AUD was associated with a higher likelihood of glaucoma diagnosis. These findings highlight the importance of greater attention to eye health among individuals with AUD and related conditions. Preventive measures could include routine glaucoma screening and open patient–physician discussions about alcohol intake. At present, glaucoma screening is not a standard component of alcohol rehabilitation programs. Incorporating vision screening and eye care into AUD treatment settings may represent an important preventive strategy to help safeguard long-term ocular health in this at-risk population. Future research should address the mechanisms linking chronic alcohol use and glaucomatous damage, including oxidative stress, vascular dysregulation, and intraocular pressure regulation. Furthermore, prospective longitudinal cohort studies are necessary to establish temporal and causal associations between AUD and glaucoma and its progression. Lastly, intervention-based studies could assess whether integrating glaucoma screenings in AUD populations improves outcomes.

## Figures and Tables

**Table 1:** Demographic and characteristics of U.S. adults (≥18 years old) in this analysis.

Participant Total in Cohort		N = 122,706
Age (mean (SD))		56.74 (16.77)
Sex at birth N (%)	Female	81,045 (66.0%)
	Male	41,661 (34.0%)
Race N (%)	Asian	3645 (3.0%)
	Black or African American	11,949 (9.7%)
	Middle Eastern or North African	709 (0.6%)
	More than one population	2285 (1.9%)
	Native Hawaiian or Other Pacific Islander	82 (0.1%)
	None Indicated	11,887 (9.7%)
	White	89,235 (72.7%)
	Missing *	2914 (2.4%)
Ethnicity N (%)	Hispanic or Latino	14,498 (11.8%)
	Not Hispanic or Latino	105,294 (85.8%)
	Missing *	2914 (2.4%)
BMI kg/m^2^ (mean (SD))		29.61 (7.41)
Glaucoma N (%)	No diagnosis	112,109 (91.4%)
	Diagnosis	10,597 (8.6%)
AUD N (%)	No diagnosis	115,152 (93.8%)
	Diagnosis	7554 (6.2%)
Smoking status N (%)	Never smoker	10,696 (8.7%)
	Ex-smoker	77,284 (63.0%)
	Current smoker	34,726 (28.3%)
Cannabis use N (%)	Never user	58,712 (47.8%)
	Ever user	63,994 (52.2%)

Footnote: * Possible answer by respondent: ‘none of these’, ‘prefer not to say’, or skipped entry; Abbreviations: BMI: Body Mass Index, AUD: Alcohol Use Disorder.

**Table 2:** Demographic Differences Between Sex.

	Level	Female	Male	*p*-Value
N (%)		81,045 (66%)	41,661 (33.9%)	
Age (mean (SD))		54.89 (16.62)	60.33 (16.46)	**<0.001 ^a^**
Race N (%)	Asian	2264 (2.8%)	1381 (3.3%)	**<0.001 ^b^**
	Black or African American	8756 (10.8%)	3193 (7.7%)	
	Middle Eastern or North African	406 (0.5%)	303 (0.7%)	
	More than one population	1612 (2.0%)	673 (1.6%)	
	Native Hawaiian or Other Pacific Islander	52 (0.1%)	30 (0.1%)	
	None Indicated	8715 (10.8%)	3172 (7.6%)	
	White	57,414 (70.8%)	31,821 (76.4%)	
	Missing *	1826 (2.3%)	1088 (2.6%)	
Ethnicity N (%)	Hispanic or Latino	10,577 (13.1%)	3921 (9.4%)	**<0.001 ^b^**
	Not Hispanic or Latino	68,642 (84.7%)	36,652 (88.0%)	
	Missing *	1826 (2.3%)	1088 (2.6%)	
BMI (kg/m^2^) (mean (SD)		29.73 (7.99)	29.38 (6.15)	**<0.001 ^a^**
Glaucoma N (%)	No diagnosis	74,745 (92.2%)	37,364 (89.7%)	**<0.001 ^b^**
	Diagnosis	6300 (7.8%)	**4297 (10.3%)**	
AUD N (%)	No diagnosis	77,599 (95.7%)	37,553 (90.1%)	**<0.001 ^b^**
	Diagnosis	3446 (4.3%)	4108 (9.9%)	
Smoking status N (%)	Never smoker	53,510 (66.0%)	23,774 (57.1%)	**<0.001 ^b^**
	Ex-smoker	20,807 (25.7%)	13,919 (33.4%)	
	Current smoker	6728 (8.3%)	3968 (9.5%)	
Cannabis user N (%)	Never user	40,423 (49.9%)	18,289 (43.9%)	**<0.001 ^b^**
	Ever user	40,622 (50.1%)	23,372 (56.1%)	

Footnote: ^a^ *t*-test; ^b^ Chi-square test; * Possible answer by respondent: ‘none of these’, ‘prefer not to say’, or skipped entry; Abbreviations: BMI: Body Mass Index, AUD: Alcohol Use Disorder. Bold indicates *p* < 0.05.

**Table 3:** Univariable logistic regression models for glaucoma diagnosis.

Term	For Categorical Variables, n for Level Shown (of 117,578 Total)	Odds Ratio	95% Confidence Interval	*p*-Value ^a^
AUD diagnosis	7225	1.479	1.374–1.591	**<0.001**
Age	Continuous	1.057	1.056–1.059	**<0.001**
Male sex (Ref = Female)	40,034	1.361	1.306–1.418	**<0.001**
BMI (kg/m^2^)	Continuous	1.000	0.998–1.003	0.843
Ex-smokers (Ref = Never smokers)	33,301	1.482	1.419–1.548	**<0.001**
Current smokers (Ref = Never smokers)	10,241	0.934	0.863–1.010	0.091
Cannabis users (Ref = Never users)	61,236	0.780	0.749–0.812	**<0.001**

Footnote: ^a^ Univariable logistic regression models. Abbreviations: BMI: Body Mass Index, AUD: Alcohol Use Disorder. Bold indicates *p* < 0.05.

**Table 4:** Multivariable logistic regression model for glaucoma diagnosis.

Term	Odds Ratio	95% Confidence Interval	*p*-Value ^a^
**AUD diagnosis**	1.455	1.345–1.572	**<0.001**
**Age**	1.058	1.056–1.060	**<0.001**
**Male sex** **(Ref = Female)**	1.030	0.986–1.075	0.183
**BMI (kg/m^2^)**	1.008	1.005–1.011	**<0.001**
**Ex-smokers** **(Ref = Never smokers)**	0.985	0.939–1.032	0.523
**Current smokers** **(Ref = Never smokers)**	1.028	0.946–1.116	0.515
**Cannabis users** **(Ref = Never users)**	0.945	0.905–0.988	**0.013**

Footnote: ^a^ Multivariable logistic regression models. Abbreviations: BMI: Body Mass Index, AUD: Alcohol Use Disorder. Bold indicates *p* < 0.05.

## Data Availability

This study used data from the All of Us Research Program’s Controlled Tier Dataset v7, available to authorized users on the Researcher Workbench.

## Supplementary Materials

The following supporting information can be downloaded at: https://www.mdpi.com/article/10.3390/ijerph22111738/s1, Supplementary Table S1. Alcohol abuse-related conditions and prevalence in the cohort; Supplementary Table S2. Glaucoma conditions and prevalence in the cohort; Supplementary Table S3. Interaction model for effect modification by sex; Supplementary Table S4A. Stratified multivariable logistic regression model (female); Supplementary Table S4B. Stratified multivariable logistic regression model (male); Supplementary Table S5A. Multivariable logistic regression model informed by only EHR-derived AUD diagnoses; Supplementary Table S5B. Multivariable logistic regression model informed by only self-reported AUD diagnoses (All of Us Personal Health History survey); Supplementary Table S6. Area under the receiver operating characteristic curve (AUC-ROC) for the multivariable logistic regression model and models informed by only survey- or EHR-derived diagnoses.

## Disclaimer

This research was supported by the Intramural Research Program of the National Institutes of Health (NIH). The contributions of the NIH authors are considered Works of the United States Government. The findings and conclusions presented in this paper are those of the author(s) and do not necessarily reflect the views of the NIH or the U.S. Department of Health and Human Services.

## Abbreviations

The following abbreviations are used in this manuscript:

AUD	Alcohol Use Disorder
AoU	All of Us
EHR	Electronic Health Record
NIH	National Institutes of Health
