# Supplementary material for: Association Between Alcohol Use Disorder and Glaucoma: Evidence from the National Institutes of Health All of Us Research Program
Source: Int J Environ Res Public Health. 2025 Nov 18;22(11):1738. doi: 10.3390/ijerph22111738 (PMC12652011; doi:10.3390/ijerph22111738)
Supplement: Supplementary file 1 [file ijerph-22-01738-s001.zip › ijerph-3893759-supplementary.pdf]

Supplementary Material

Supplementary Table S1. Alcohol abuse-related conditions and prevalence in the cohort

**Alcohol related diagnoses- EHR Conditions**	**N (%) of 4,923**
Alcohol abuse	3600 (73.1%)
Alcohol dependence	2307 (46.9%)
Chronic alcoholism in remission	1080 (21.9%)
Alcohol withdrawal syndrome	437 (8.88%)
Alcoholic cirrhosis	349 (7.09%)
Continuous chronic alcoholism	292 (5.93%)
Alcoholic fatty liver	243 (4.94%)
Acute alcoholic intoxication in alcoholism	237 (4.81%)
Alcoholic liver damage	168 (3.41%)
Alcoholic hepatitis	159 (3.23%)
Alcohol withdrawal delirium	93 (1.89%)
Acute alcoholic liver disease	85 (1.73%)
Continuous acute alcoholic intoxication in alcoholism	72 (1.46%)
Episodic chronic alcoholism	71 (1.44%)
Alcohol-induced chronic pancreatitis	65 (1.32%)
Alcoholic hepatic failure	45 (0.914%)
Persistent alcohol abuse	37 (0.752%)
Alcoholism	31 (0.63%)
Severe alcohol dependence	19 (0.386%)
Moderate alcohol dependence	16 (0.325%)
Dementia associated with alcoholism	14 (0.284%)
Episodic acute alcoholic intoxication in alcoholism	12 (0.244%)
Acute alcoholic intoxication in remission, in alcoholism	11 (0.223%)
Alcohol dependence in pregnancy	11 (0.223%)
Alcohol induced disorder co-occurrent and due to alcohol dependence	10 (0.203%)
Alcoholic fibrosis and sclerosis of liver	6 (0.122%)

Supplementary Table S2. Glaucoma conditions and prevalence in the cohort

Glaucoma EHR Conditions	N (%) of 8,199
Borderline glaucoma	3691 (45%)
Glaucoma	3018 (36.8%)
Open-angle glaucoma - borderline	1888 (23%)
Primary open angle glaucoma	1683 (20.5%)
Open angle with borderline intraocular pressure	1531 (18.7%)
Glaucomatous atrophy of optic disc	929 (11.3%)
Open-angle glaucoma	690 (8.42%)
Glaucoma suspect	588 (7.17%)
Anatomical narrow angle glaucoma with borderline intraocular pressure	574 (7%)
Low tension glaucoma	327 (3.99%)
At risk of glaucoma	179 (2.18%)
Primary angle-closure glaucoma	168 (2.05%)
Glaucoma associated with ocular disorder	156 (1.9%)
Pseudoexfoliation glaucoma	127 (1.55%)
Pigmentary glaucoma	107 (1.31%)
Open-angle glaucoma of left eye	70 (0.854%)
Open-angle glaucoma of right eye	68 (0.829%)
Acute angle-closure glaucoma	51 (0.622%)
Bilateral primary open angle glaucoma	34 (0.415%)
Bilateral low-tension glaucoma of eyes	23 (0.281%)
Intermittent angle-closure glaucoma	15 (0.183%)
Bilateral glaucoma	13 (0.159%)
Low tension glaucoma of left eye	9 (0.11%)
Bilateral eye anatomic narrow angle glaucoma	8 (0.0976%)
Low tension glaucoma of right eye	8 (0.0976%)
Angle-closure glaucoma	7 (0.0854%)
Bilateral angle-closure glaucoma	6 (0.0732%)
Bilateral open-angle glaucoma	6 (0.0732%)
Primary open angle glaucoma of right eye	5 (0.061%)
Glaucoma of right eye	4 (0.0488%)
Bilateral pseudoexfoliation glaucoma of eyes	3 (0.0366%)
Pseudoexfoliation glaucoma of left eye	3 (0.0366%)
Glaucoma of left eye	2 (0.0244%)
Primary open angle glaucoma of left eye	2 (0.0244%)
Pseudoexfoliation glaucoma of right eye	2 (0.0244%)
Anatomic narrow angle glaucoma of left eye	1 (0.0122%)
Bilateral pigmentary glaucoma of eyes	1 (0.0122%)

Supplementary Table S3. Interaction model for effect modification by sex

Term	Coefficient estimate	Standard error	Statistic (z)	*p*-value[a]
**(Intercept)**	-6.099	0.081	-75.144	**<0.001**
**Male sex (Ref = Female)**	0.0320	0.023	1.387	0.166
**AUD**	0.393	0.061	6.484	**<0.001**
**Age**	0.056	0.001	63.011	**<0.001**
**BMI**	0.008	0.002	5.194	**<0.001**
**Ex-smokers (Ref = Never smokers)**	-0.016	0.024	-0.643	0.520
**Current smokers (Ref = Never smokers)**	0.027	0.042	0.646	0.518
**Cannabis users (Ref = Never users)**	-0.056	0.023	-2.493	**0.013**
**Sex*AUD interaction**	-0.031	0.079	-0.391	0.696

Footnote: [a]Multivariable logistic regression model. Abbreviations: BMI: Body Mass Index, AUD: Alcohol Use Disorder. Bold indicates $p<.05$.

Supplementary Table S4A. Stratified multivariable logistic regression model (female)

Term	Odds Ratio	95% Confidence Interval	*p*-value[a]
**(Intercept)**			**<0.001**
**AUD**	1.496	1.326 - 1.684	**<0.001**
**Age**	1.057	1.055 - 1.060	**<0.001**
**BMI**	1.007	1.004 - 1.011	**<0.001**
**Ex-smokers (Ref = Never smokers)**	0.983	0.923 - 1.046	0.588
**Current smokers (Ref = Never smokers)**	1.057	0.948 - 1.177	0.313
**Cannabis users (Ref = Never users)**	0.893	0.843 - 0.946	**<0.001**

Footnote: [a]Multivariable logistic regression model. Abbreviations: BMI: Body Mass Index, AUD: Alcohol Use Disorder. Bold indicates $p<.05$.

Supplementary Table S4B. Stratified multivariable logistic regression model (male)

Term	Odds Ratio	95% Confidence Interval	*p*-value[a]
**(Intercept)**			**<0.001**
**AUD**	1.418	1.278 - 1.570	**<0.001**
**Age**	1.059	1.056 - 1.062	**<0.001**
**BMI**	1.009	1.004 - 1.015	**<0.01**
**Ex-smokers (Ref = Never smokers)**	0.992	0.922 - 1.066	0.823
**Current smokers (Ref = Never smokers)**	0.995	0.873 - 1.131	0.941
**Cannabis users (Ref = Never users)**	1.029	0.960 - 1.103	0.416

Footnote: [a]Multivariable logistic regression model. Abbreviations: BMI: Body Mass Index, AUD: Alcohol Use Disorder. Bold indicates *p*<.05.

Supplementary Table S5A. Multivariable logistic regression model informed by only EHR-derived AUD diagnoses.

Term	Odds Ratio	95% Confidence Interval	*p*-value[a]
**Intercept**			**<0.001**
**AUD (EHR)**	1.695	1.548 - 1.853	**<0.001**
**Age**	1.058	1.056 - 1.060	**<0.001**
**Male sex (Ref = Female)**	1.029	0.986 - 1.075	0.189
**BMI**	1.008	1.005 - 1.011	**<0.001**
**Ex-smokers (Ref = Never smokers)**	0.990	0.944 - 1.037	0.666
**Current smokers (Ref = Never smokers)**	1.022	0.941 - 1.110	0.601
**Cannabis users (Ref = Never users)**	0.949	0.908 - 0.992	**0.020**

Footnote: [a]Multivariable logistic regression model. Abbreviations: BMI: Body Mass Index, AUD: Alcohol Use Disorder. Bold indicates *p*<.05.

Supplementary Table S5B. Multivariable logistic regression model informed by only self-reported AUD diagnoses (All of Us Personal Health History survey).

Term	Odds Ratio	95% Confidence Interval	*p*-value[a]
**Intercept**			**<0.001**
**AUD (survey)**	1.223	1.099 - 1.356	**<0.001**
**Age**	1.058	1.056 - 1.060	**<0.001**
**Male sex (Ref = Female)**	1.045	1.001 - 1.091	**0.0441**
**BMI**	1.008	1.005 - 1.011	**<0.001**
**Ex-smokers (Ref = Never smokers)**	0.999	0.953 - 1.047	0.964
**Current smokers (Ref = Never smokers)**	1.071	0.986 - 1.162	0.101
**Cannabis users (Ref = Never users)**	0.953	0.912 - 0.997	**0.034**

Footnote: [a]Multivariable logistic regression model. Abbreviations: BMI: Body Mass Index, AUD: Alcohol Use Disorder. Bold indicates p<.05.

Supplementary Table S6. Area under the receiver operating characteristic curve (AUC-ROC) for the multivariable logistic regression model and models informed by only survey- or EHR-derived diagnoses.

Model	AUC-ROC	95% Confidence Interval (DeLong)
**Main model**	0.720	0.716 - 0.725
**Survey (self-reported AUD diagnoses)**	0.719	0.714 - 0.724
**EHR-derived AUD diagnoses**	0.721	0.716 - 0.726
